# Oxidative stress resistance prompts pyrroloquinoline quinone biosynthesis in *Hyphomicrobium denitrificans* H4-45

**DOI:** 10.1007/s00253-024-13053-1

**Published:** 2024-02-13

**Authors:** Jiale Liang, Mingjie Tang, Lang Chen, Wenjie Wang, Xinle Liang

**Affiliations:** School of Food Science and Biotechnology, Zhejiang Gongshang University, Hangzhou, 310018 China

**Keywords:** Pyrroloquinoline quinone, *Hyphomicrobium denitrificans*, Adaptive laboratory evolution, Random mutagenesis, Whole-genome sequencing analysis

## Abstract

**Abstract:**

Pyrroloquinoline quinone (PQQ) is a natural antioxidant with diverse applications in food and pharmaceutical industries. A lot of effort has been devoted toward the discovery of PQQ high-producing microbial species and characterization of biosynthesis, but it is still challenging to achieve a high PQQ yield. In this study, a combined strategy of random mutagenesis and adaptive laboratory evolution (ALE) with fermentation optimization was applied to improve PQQ production in *Hyphomicrobium denitrificans* H4-45. A mutant strain AE-9 was obtained after nearly 400 generations of UV-LiCl mutagenesis, followed by an ALE process, which was conducted with a consecutive increase of oxidative stress generated by kanamycin, sodium sulfide, and potassium tellurite. In the flask culture condition, the PQQ production in mutant strain AE-9 had an 80.4% increase, and the cell density increased by 14.9% when compared with that of the initial strain H4-45. Moreover, batch and fed-batch fermentation processes were optimized to further improve PQQ production by pH control strategy, methanol and H_2_O_2_ feed flow, and segmented fermentation process. Finally, the highest PQQ production and productivity of the mutant strain AE-9 reached 307 mg/L and 4.26 mg/L/h in a 3.7-L bioreactor, respectively. Whole genome sequencing analysis showed that genetic mutations in the *ftfL* gene and *thiC* gene might contribute to improving PQQ production by enhancing methanol consumption and cell growth in the AE-9 strain. Our study provided a systematic strategy to obtain a PQQ high-producing mutant strain and achieve high production of PQQ in fermentation. These practical methods could be applicable to improve the production of other antioxidant compounds with uncleared regulation mechanisms.

**Key points:**

*• Improvement of PQQ production by UV-LiCl mutagenesis combined with adaptive laboratory evolution (ALE) and fermentation optimization.*

*• A consecutive increase of oxidative stress could be used as the antagonistic factor for ALE to enhance PQQ production.*

*• Mutations in the ftfL gene and thiC gene indicated that PQQ production might be increased by enhancing methanol consumption and cell growth.*

**Supplementary information:**

The online version contains supplementary material available at 10.1007/s00253-024-13053-1.

## Introduction

Pyrroloquinoline quinone (PQQ), a tricyclic ortho-quinone compound, was reported as a cofactor for universal redox dehydrogenases, including glucose dehydrogenase, alcohol dehydrogenase, and methanol dehydrogenase (Anthony 2001; Dai et al. 2022; Okuda and Sode 2004). PQQ has radical scavenging activity and is appreciated as a natural antioxidant (Alkahtani et al. 2021). It has been reported that PQQ is involved in a variety of biological processes, including neuroprotection (Shanan et al. 2019; Wen et al. 2020), nephroprotection (Jonscher et al. 2021), anti-diabetic (Kumar and Kar 2015; Qu et al. 2022), and protection of the cardiac mitochondria from damage (Nehra et al. 2017; Xu et al. 2020). It is known that PQQ is contained in different kinds of food and beverages such as tofu, papaya, and fermented natto (Kumazawa et al. 1995). The absence of PQQ from nutrient sources produces an abnormality like a vitamin-related deficiency, so PQQ is considered as a “longevity vitamin” (Jonscher et al. 2021). Therefore, a sufficient supply of PQQ is required due to its wide applications and growing demands in the food and pharmaceutical industry.

A certain amount of PQQ production has been shown in some methanol-utilizing microorganisms, such as *Methylobacterium* sp. (Si et al. 2017, 2016; Wei et al. 2017), *Methylovorus* sp. (Xiong et al. 2011), and *Hyphomicrobium denitrificans* (Liu et al. 2020; Urakami et al. 1992). Methanol dehydrogenase (MDH) in methylotrophic bacteria catalyzes the first step of methanol utilization, and PQQ serves as a crucial coenzyme for MDH (Keltjens et al. 2014). Among them, *H. denitrificans* showed great potential for PQQ production and has been used as the only industrial strain to produce PQQ by several biopharmaceutical companies (Ren et al. 2023). However, the concentration and productivity of PQQ were relatively low in the wild strain.

Various random mutagenesis approaches and adaptive laboratory evolution (ALE) strategies have been used to obtain mutations of interest with the potential of only single nucleotide changes (Bose 2016). UV and chemical mutagenesis were commonly used to generate mutations with a selectable trait. Instead of loss-of-function mutations, both chemical and UV mutagenesis have the potential to generate gain-of-function mutations that can yield tremendous insight. For example, a single nucleotide change in an enzyme may alter the specificity of its substrate or make it constitutively active (Bose 2016). An easy selection process is needed after random mutagenesis. ALE is an increasingly popular technique for applying pressure on a microbial population to promote the acquisition of mutations that will improve cell fitness (Sandberg et al. 2019). A combined strategy composing mutagenesis approaches and ALE has improved the production of PQQ (Ren et al. 2023) and many other compounds, including astaxanthin (Jiang et al. 2020), 1,3-propanediol (Yun et al. 2022), 4-hydroxyphenylacetic acid (Shen et al. 2022b), and S-adenosyl-L-methionine (Weng et al. 2022). The mutations and selections are important due to the potential of breaking the limits of increasing PQQ productivity and could obtain an inherent property of genetic material. However, little is known about the single nucleotide changes after mutagenesis and the ALE process in *H. denitrificans*, which hinders the way to identify critical proteins that involved in the regulation of PQQ production.

In this study, a combined mutagenesis strategy with UV-based mutagenesis and LiCl mutagenesis followed by ALE was carried out with our isolated strain *Hyphomicrobium denitrificans* H4-45 to obtain a high-titer PQQ mutant strain. Due to the antioxidant properties of PQQ, a consecutive increase of oxidative stress generated by kanamycin, sodium sulfide, and potassium tellurite was used as the antagonistic factor for ALE. Kanamycin is known as an aminoglycoside antibiotic agent which could bind to the bacterial 30S ribosomal subunit to inhibit protein synthesis and could also stimulate the formation of reactive oxygen species (ROS) in both enzymatic and non-enzymatic superoxide-generating systems (Sha and Schacht 1999). Sodium sulfide (Na_2_S) is a fast-releasing H_2_S donor, which induces DNA damage by increasing oxidative stress (Xiao et al. 2019). And the toxicity of potassium tellurite (K_2_TeO_3_) also involves superoxide formation (Pérez et al. 2007). Afterward, to further increase the PQQ concentration, the optimization of fermentation was conducted by using a pH control strategy, adding methanol feed flow and appropriate H_2_O_2_, and splitting the fermentation process. Moreover, the whole genome resequencing analyses of the mutant strain and parental strain were performed to provide insights into the mechanisms involved in the increase of PQQ production in *H. denitrificans*.

## Material and methods

### Strains and media

The *Hyphomicrobium denitrificans* H4-45 strain (CCTCC NO. M2019284) was isolated from Rice Wine grains in our laboratory and preserved in the China Center for Type Culture Collection. All strains used in this study were derivative mutants of the wild-type and listed in Supplementary Table S1. All strains were stored at − 80 °C in 20% glycerol.

The MM medium contains 20 mL/L methanol, 3 g/L (NH_4_)_2_SO_4_, 1.4 g/L KH_2_PO_4_, 3 g/L Na_2_HPO_4_, 1 g/L MgSO_4_·7H_2_O, and 1.0 mL/L trace element solution, and the pH of this medium was adjusted to 7.0 using 5 M sodium hydroxide. The trace element solution contains 7.5 g/L FeSO_4_·7H_2_O, 4.5 g/L MnSO_4_·5H_2_O, 0.75 g/L CuSO_4_·5H_2_O, 1.5 g/L NaCl, 30 mg/L (NH_4_)_6_Mo_7_O_24_·4H_2_O, 30 mg/L KI, 30 mg/L CoCl_2_·6H_2_O, and 30 mg/L H_3_BO_3_. For solid plates, 2.0% agar was supplemented.

General culture and fermentation experiments of cells were done with MM medium. For the ALE medium, kanamycin (3–30 mg/L), sodium sulfide (3–9 mg/mL), or potassium tellurite (20–60 mg/L) were added before inoculation to provide a stress condition as required.

### UV‑LiCl mutagenesis and adaptive laboratory evolution

*H. denitrificans* H4-45 was treated by UV-LiCl mutagenesis as previously described (Zhang et al. 2014). Briefly, to establish the optimal conditions for UV mutagenesis, 5 mL of cell suspension was transferred to a sterile plate (9 cm in diameter) on a magnetic stirrer and irradiated at 30 cm directly below a UV light (30 W) for different times. After mutagenesis, the suspension was washed with 6-mL sterile 0.9% NaCl, maintained at 4 °C in the dark for 2 h, then diluted to the appropriate concentration and coated evenly on an MM agar plate at 30 °C in the dark for 7 d. The mortality rate was obtained from the following Eq. (1), and the mortality rate curve was examined (Supplementary Fig. S1). *H.denitrificans* H4-45 was first treated with UV for an appropriate time, and 5% (v/v) culture was transferred to a new medium with 0.6% LiCl and cultured to the logarithmic phase. The mutagenized population was used as the initial cell pool for evolving mutants by each round of ALE.

1 $$\mathrm{Mortality\ rate }\mathrm{=}\frac{\mathrm{No. of colonies(control) - No. of colonies(treatment)}}{\mathrm{No. of colonies (control)}} \times 100\mathrm{\%}$$

Adaptive laboratory evolution was performed by three rounds of sequential transfers with a working volume of 30 mL in a 250-mL shake-flask at 200 rpm and 30 ℃. A robust growth-coupling strategy is a prerequisite for the success of ALE (Wu et al. 2022). Before each round of evolution, different concentrations of screening agents were directly added to the medium to determine the initial screening pressure concentration for each round of ALE, and the growth kinetics was measured. The initial concentrations of kanamycin, sodium sulfide, and potassium tellurite were determined as 3 mg/L, 3 mg/mL, and 20 mg/L, respectively (Supplementary Fig. S2). According to the growth situation, 5% (v/v) cells mutated by UV-LiCl were transferred to a fresh medium (initial OD_600_ of 0.2) containing different concentrations of kanamycin (3 mg/L, 15 mg/L, 30 mg/L) in turn. After OD_600_ did not change much for at least three passages of repeating cultivation, the best-performing isolate was selected for the next stage of the starting strain. In the second and third rounds of evolution, different concentrations of sodium sulfide (3 mg/mL, 6 mg/mL, 9 mg/mL) and potassium tellurite (20 mg/L, 40 mg/L, 60 mg/L) were superimposed on the basis of the previous screening pressure. Finally, the mutant strain with the highest PQQ production was detected for genetic stability after 9 passages and selective pressure tolerance. Before each transfer, the cultures were frozen with glycerol and stored at − 80 ℃ at the same screening agent concentration.

### Isolating mutants from evolved populations

For cultivation, cells stored at − 80 ℃ were spread onto medium plates and incubated at 30 ℃ for seven to ten days. Because PQQ generated a reddish-brown appearance on colonies, the colorimetrical method was used to screen the library. Single colonies with a reddish-brown appearance and bigger size were picked up and transferred to a 30-mL MM medium. After being cultured for 48 h at 200 rpm and 30 ℃, 1-mL seed culture was inoculated into a 500-mL shake-flask with 80-mL medium and then incubated at 30 ℃ for 120 h. After incubation, the cultures were analyzed for cell density and PQQ content. The best-performing isolate from evolution was selected for further analysis.

### Whole-genome sequencing and mutation identification

Genomic DNA was extracted using Bacteria DNA Kit (OMEGA) according to the manufacturer’s instructions. A high-qualified DNA sample was used to construct the fragment library. Whole-genome sequencing was performed on the Illumina NovaSeq 6000 sequencing (Shanghai BIOZERON Co., Ltd.). The raw paired-end reads were trimmed and quality controlled by Trimmomatic with default parameters. ABySS (version 2.3.4) was used to do genome assembly with multiple-Kmer parameters and got the optimal results of the assembly. GapCloser software (version 1.12) was applied to fill the remaining local inner gaps and correct the single base polymorphism for the final assembly results. The genomes were annotated, and genes were categorized by protein functions using the Rapid Annotations Subsystems Technology (RAST) server with version 2.0 (Aziz et al. 2008). The paired-end reads from strain AE-9 were aligned to the reference genome of strain H4-45 using the BWA software (version 0.7.17) (Jung and Han 2022). Potential mutations including single nucleotide variations and insertion/deletions were identified using the SAMtools software (version 1.15) (Li et al. 2009) and BCFtools software (version 1.15) (Danecek and McCarthy 2017). Multiple sequence alignment and annotation of the detected variants were conducted using Geneious Prime (version 2022.0.4). The raw sequencing data in this study was submitted to BioProject (accession no. PRJNA1002585) on the NCBI.

### Batch and fed-batch production of PQQ in 3.7 L bioreactor

Batch and fed-batch fermentation processes were conducted in a 3.7-L bioreactor (Bioengineering AG, Wald, Switzerland). A hundred microliter glycerol-stock of the *H. denitrificans* AE-9 was inoculated into 30-mL MM medium and cultured at 30 °C, 200 rpm for 48 h. Then, the preculture was transferred to a 200-mL fresh MM medium and grew until entering the mid-exponential phase. Ten percent (v/v) seed culture was transferred to a 1.8-L MM medium in a 3.7-L bioreactor. The fermentation was carried out at 30 °C. The pH was maintained at 6 by automatically feeding 5 M sodium hydroxide. The agitation speed was 450 rpm, and the air was supplied at 1.25 vvm. For fed-batch fermentation, after the initial was below 4 mL/L, 300 mL/L methanol solution was supplemented to maintain the methanol concentration. For the H_2_O_2_ feeding experiment, at the same time as methanol feeding, add 100 mL of hydrogen peroxide at 200 mM, 400 mM, and 600 mM per 4 h.

For segmented fermentation, seed culture (200 mL) inoculated the 3.7-L bioreactor with MM medium (1.8 L) and cultured for 48 h. The fermentation broth was divided according to different proportions after batch fermentation culture, and the segmentation levels were 10%, 20%, and 30%, respectively. Then, fresh MM medium was added to 2 L, and the rest continued to be cultured for 96 h. Segmentation fermentation was also conducted with the methanol and H_2_O_2_ feeding process designed above. The fermentation broth was sampled every 4 h to examine PQQ, residual methanol, pH, and cell growth.

### Cell density, PQQ quantification, and methanol quantification

The cell density was measured by the absorbance under 600 nm (OD_600_) with a UV-2550 UV/Vis spectrophotometer (Shimadzu, Japan).

The calculation of the number of generations of evolution is described previously (Bentley et al. 2020). Briefly, in the logarithmic growth phase of the strain, it is assumed that the initial OD_600_ value of the bacteria was *N*_1_ at *t*_1_, and the final OD_600_ value of the bacteria was *N*_2_ at *t*_2_ after *n* divisions. Generation time and the number of generations were obtained from the following equations:

2 $${N}_{2}={N}_{1}\times {2}^{n}$$

3 $$\mathrm{The number of generations} = \frac{{\mathrm{lg}}{\mathrm{N}}_{2}-{\mathrm{lg}}{\mathrm{N}}_{1}}{\mathrm{lg2}}$$

4 $$\mathrm{Generation time}\mathrm{=}\frac{{\mathrm{t}}_{2}-{\mathrm{t}}_{1}}{\mathrm{n}}$$

The PQQ in the fermentation broth was quantified by high-performance liquid chromatography (HPLC) as in the previous study (Si et al. 2016). Briefly, the fermentation supernatant samples were filtered with a 0.22-μm organic filter membrane, and a 20.0-μL injection volume was used in a mobile phase composed of a 75: 25 ratio of methanol to ddH_2_O with 0.1% phosphoric acid with a flow rate of 0.5 mL/min. The column temperature was maintained at 25 ℃, and UV–Vis absorption was measured at 254 nm. The PQQ concentration was calculated corresponding to the peak area according to a standard curve (Supplementary Fig. S3). The PQQ standard was purchased from Shanghai Yuanye Bio-Technology Co., Ltd.

The gas chromatography (GC) analysis of methanol was performed as in the previous study (Si et al. 2017). Briefly, a sample (1 µL) in the organic phase was injected with nitrogen as the carrier gas, and the oven temperature was heated at 80 ℃ for 1.5 min, then increased to 140 ℃ at 30 ℃/min, and was increased again to 240 ℃ at 40 ℃/min, then held for 2 min. The injector temperature was 240 ℃ with a split ratio of 20:1, and the flame ionization detector (FID) temperature was 260 ℃.

### Statistical analysis

All experiments were performed with at least three biological replicates. The data are presented as the mean ± SD, and statistical comparisons between groups were tested using Student’s *t* test. Differences were considered statistically significant at *p < 0.05, **p < 0.01, and ***p < 0.001.

## Results

### Achievement of a high PQQ-producing strain AE-9 by UV-LiCl mutagenesis combined with ALE

**Fig. 1:**
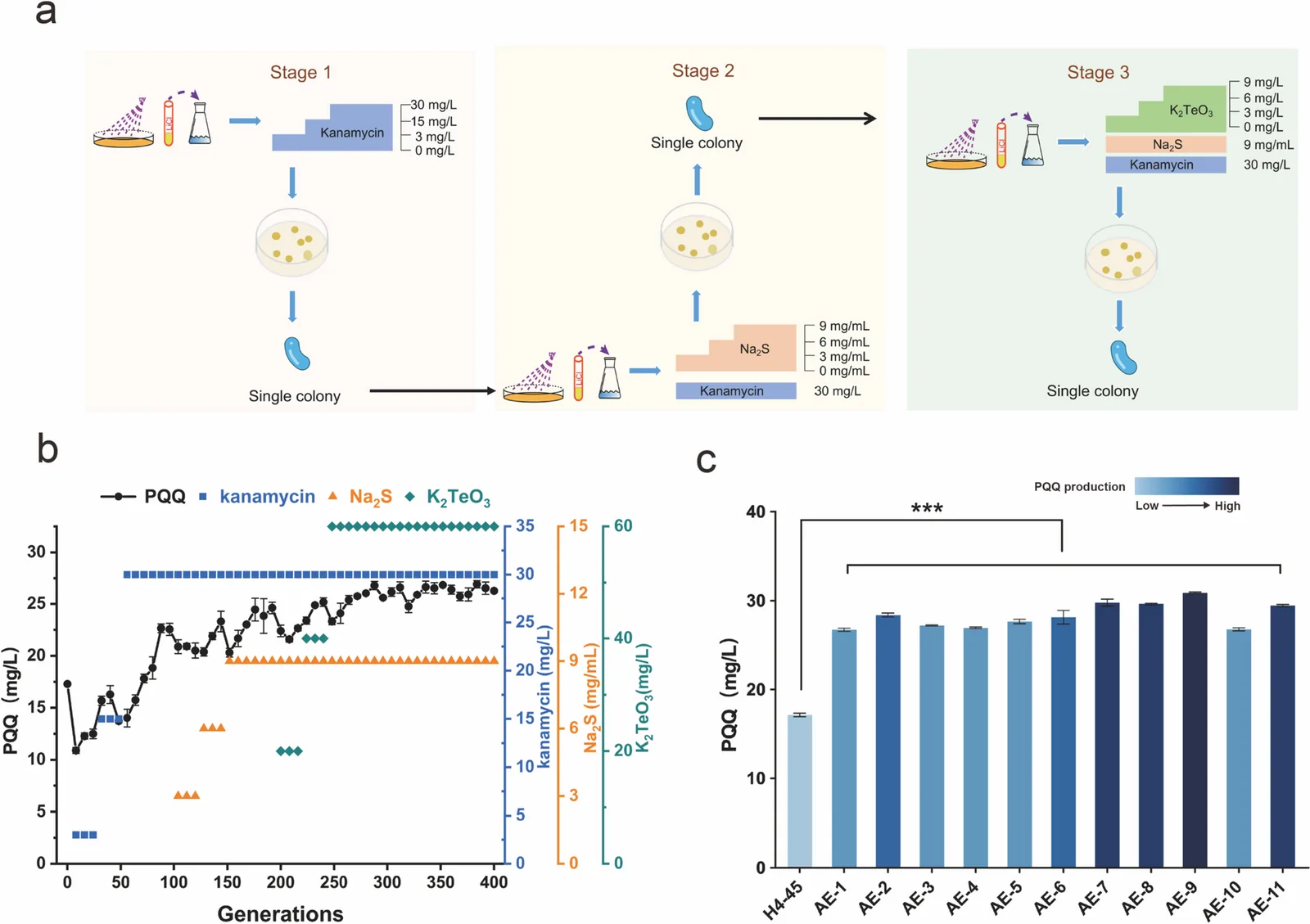
The combined strategy of UV-LiCl mutagenesis and ALE for screening PQQ high-yielding mutants. (**a**) A schematic diagram of the combined strategy performed in this study. (**b**) The trend of PQQ production during three rounds of ALE. (**c**) PQQ production of selected strains after the third round of UV-LiCl mutagenesis and ALE

The wild strain *H. denitrificans* H4-45 was isolated by our lab and showed PQQ productivity of 17.1 mg/L. To improve the production of PQQ, a combined screening system of UV-LiCl mutagenesis and adaptive laboratory evolution (ALE) was established in this study (Fig. 1a). The wild strain *H. denitrificans* H4-45 was first subjected to a UV-LiCl mutagenesis system under the different UV treatment time (30 s, 60 s, 90 s, 120 s, 150 s, 180 s). The UV exposure time was then set as 120 s with a survival rate of 20% for the following UV-LiCl mutagenesis (Supplementary Fig. S1), which is considered appropriate and effective for collecting mutants (Zhang et al. 2014). After UV-LiCl mutagenesis, the mutants were selected for an ALE process growing under a consecutive increase of oxidative stress (kanamycin, sodium sulfide, and potassium tellurite) in the fermentation medium (Fig. 1a).

Three iterative UV-LiCl-ALE selections were conducted in total. After the first round of UV-LiCl mutagenesis, the cells were collected and cultured in the MM medium with an increase of kanamycin concentration ranging from 3 mg/L, 15 mg/L to 30 mg/L for ALE. Then the mutant population was screened by measuring their PQQ productions, and the mutant strain AC-6 was selected with the PQQ content reached 23.6 mg/L (Fig. 1b, Supplementary Fig. S4). The mutant AC-6 was then selected as the starting strain for the second round of the UV-LiCl-ALE process. The UV-LiCl mutagenesis was performed using the method mentioned above. After the second round of UV-LiCl mutagenesis, 30 mg/L kanamycin combined with a consecutive increase of sodium sulfide concentration (3 mg/mL, 6 mg/mL, 9 mg/mL) was used for ALE. Then the mutant strain AD-17 was selected for the next round of UV-LiCl-ALE with the PQQ production of 27.6 mg/L (Supplementary Fig. S5). After the third round of UV-LiCl mutagenesis, ALE was conducted with 30 mg/L kanamycin, 9 mg/mL sodium sulfide, and a consecutive increase of potassium tellurite concentration (20 mg/L, 40 mg/L, 60 mg/L). Then 11 mutant strains with the highest values at OD_600_ were selected for fermentation in shake flasks to verify their PQQ concentrations (Fig. 1c). As a result, the mutant strain AE-9 with reliable stability after 51 transfers (~ 400 generations) displayed the highest PQQ production, and the PQQ content reached 30.9 ± 0.56 mg/L (Supplementary Fig. S6), which led to an 80.4% increase when compared to the wild strain H4-45. The mutant strain AE-9 was then tested genetically stable after 9 generations (Supplementary Fig. S6) and was selected for further analysis.

### Physiological characterization of the UV-LiCl-ALE derived strain AE-9

**Fig. 2:**
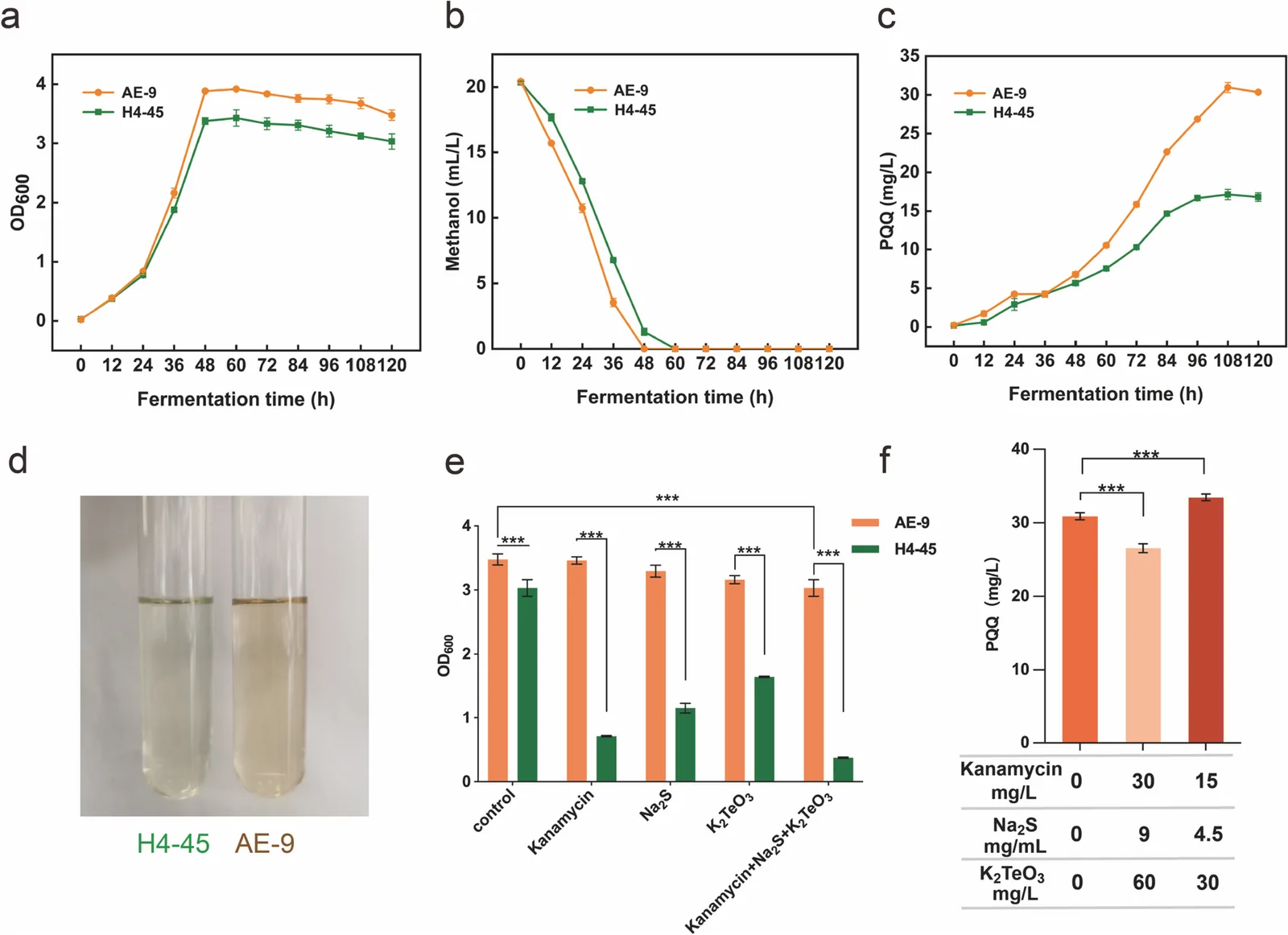
Characteristic analyses of the wild strain H4-45 and the mutant strain AE-9. The cell growth (**a**), methanol consumption (**b**), and PQQ production (**c**) of the two strains cultured in MM medium. (**d**) Visual comparison of the culture color between the wild strain H4-45 and the mutant strain AE-9. (**e**) Growth of H4-45 and AE-9 in the MM medium and the MM medium with different oxidative stress conditions (30 µg/mL kanamycin, 9 µg/mL sodium sulfide, 60 µg/mL potassium tellurite, or mixture of all). (**f**) PQQ production of mutant strain AE-9 in the MM medium with no pressure, full concentration of kanamycin + Na_2_S + K_2_TeO_3_ treatment, and half concentration of kanamycin + Na_2_S + K_2_TeO_3_ treatment. The data are presented as the means with standard deviation (SD) of three replicates. ***P < 0.001

The mutant strain AE-9 and wild strain H4-45 were first cultured on an MM medium to analyze the cell growth, methanol consumption, and PQQ production. Both strains grew well, and the OD_600_ growth of AE-9 increased above 14.9% compared to the wild strain H4-45 (Fig. 2a). Besides, the mutant strain AE-9 showed a faster methanol consumption tendency than the wild strain (Fig. 2b). After 108 h of fermentation, the concentration of PQQ reached 31.0 ± 0.65 mg/L in the mutant strain AE-9, while the wild strain produced only 17.1 ± 0.66 mg/L PQQ (Fig. 2c), which led to an observative reddish color in the AE-9 fermentation culture (Fig. 2d).

To evaluate the tolerance of oxidative stress, the mutant strain AE-9 and wild strain H4-45 were then cultured on MM medium supplemented with 30 mg/L kanamycin, 9 mg/mL Na_2_S, 60 mg/L K_2_TeO_3_, or a mixture of three all, respectively. It was noticeable that the mutant strain had comparable growth under three different oxidative stress conditions with the no pressure condition and only had a 14.4% decrease when cultured in MM + kanamycin + Na_2_S + K_2_TeO_3_ medium (Fig. 2e). But the wild strain could barely grow under pressure (Fig. 2e). We then found that PQQ production of the mutant strain also decreased 14.1% in MM + kanamycin + Na_2_S + K_2_TeO_3_ medium compared to no pressure MM medium (Fig. 2f), which indicates PQQ yield is closely related to the cell growth. Furthermore, to eliminate the growth defect in oxidative stress-inducing conditions, MM medium with a half concentration of kanamycin + Na_2_S + K_2_TeO_3_ treatment was used to culture the mutant strain AE-9. To our surprise, when cultured in a half concentration of treatment, the mutant strain AE-9 showed a 6.4% increase in cell density and an 8.4% increase in PQQ production when compared to no pressure condition, and a 26.1% increase in cell density and a 22.1% increase of PQQ production when compared to the full concentration of treatment (Fig. 2f, Supplementary Fig. S7). It indicates that fermentation with appropriate oxidative stress might induce higher PQQ production and be useful for the following fermentation optimization. As a result, the mutant strain AE-9 had a higher capability of PQQ productivity than the wild strain and obtained the ability to tolerate oxidative stress generated by kanamycin + Na_2_S + K_2_TeO_3_ treatment.

### PQQ production in batch and fed-batch fermentation

**Fig. 3:**
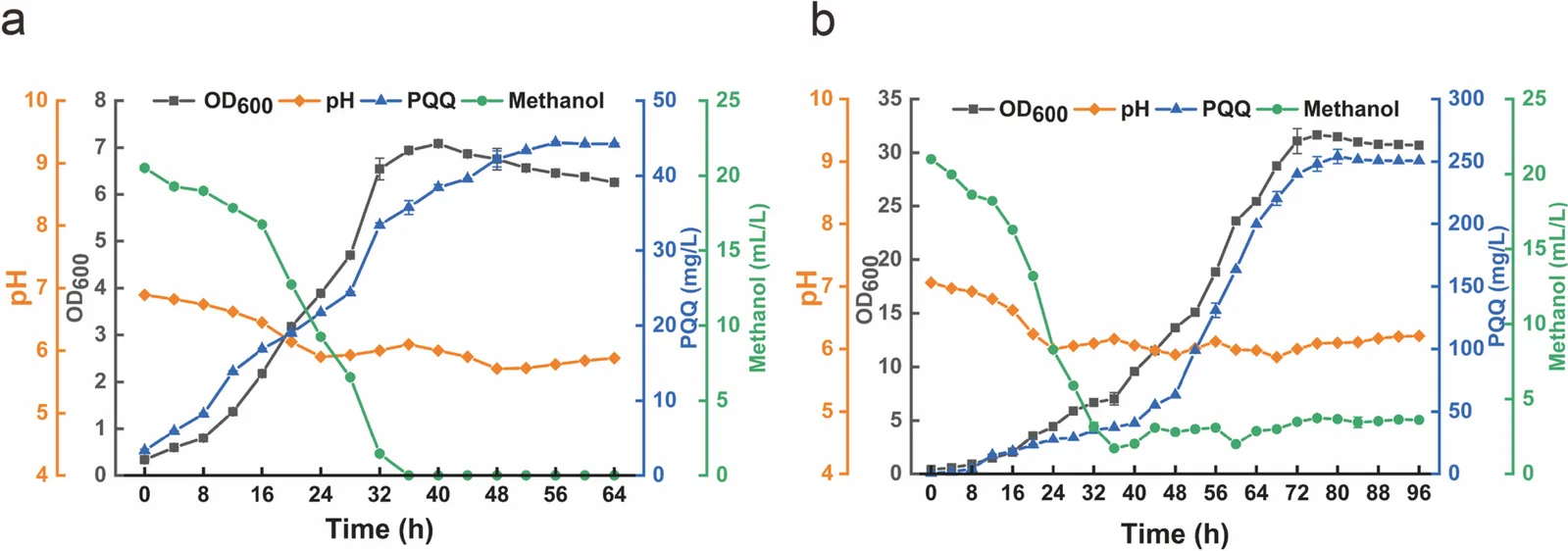
Optimizing batch (**a**) and fed-batch (**b**) fermentation of PQQ production in a 3.7-L bioreactor by pH control strategy and methanol feed flow

The batch fermentation of the mutant strain *H. denitrificans* AE-9 was carried out in a 3.7-L fermenter with an initial pH of 7.0. The cell growth, PQQ production, pH values, and methanol consumption were analyzed during the whole process. It was noticed that the pH was decreasing during the fermentation process and then maintained at 5.0 after 32 h (Supplementary Fig. S8). It has been reported that pH 5.0 is not beneficial for either cell growth or PQQ production (Si et al. 2017). To remove the pH limitation on PQQ production, a two-stage pH control strategy was conducted with an initial pH of 7.0 and maintained at a constant pH of 6.0 later (Fig. 3a). Compared to the pH-uncontrolled fermentation, the pH two-stage process showed 21% increase of PQQ production and reached 44.4 mg/L.

Methanol was reported to be utilized by *H. denitrificans* as a single carbon source (Anthony 1986). To promote both cell growth and PQQ accumulation in AE-9, fed-batch fermentation was performed by combining a methanol feed flow. The initial methanol concentration was 20 mL/L, then a constant feeding of methanol was applied after the initial methanol was consumed completely to keep the methanol concentration maintained at 4 mL/L. The PQQ production reached 254 mg/L after 80 h of fermentation, while the cell densities increased to OD_600_ 31.7 at 76 h (Fig. 3b).

### Further improvement of PQQ production by optimizing H2O2 and segmented fermentation

**Fig. 4:**
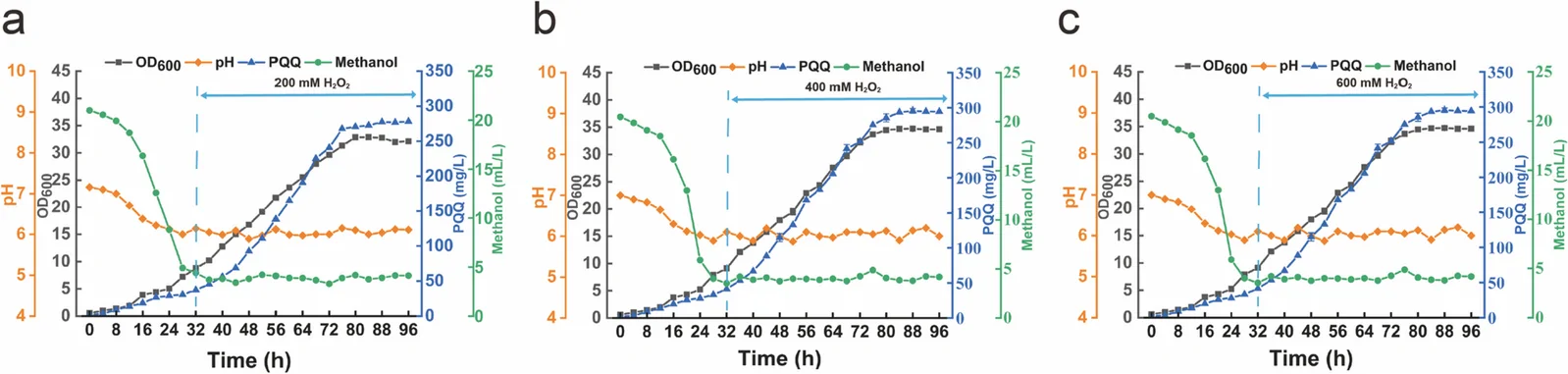
Effects of different H_2_O_2_ treatments on the kinetic parameters during fed-batch fermentation in a 3.7-L bioreactor. Modes A (**a**), B (**b**), and C (**c**) represent the addition of 100 mL H_2_O_2_ at a concentration of 200 mM, 400 mM, and 600 mM per 4 h for 32–96 h, respectively

To explore the potential of oxidative stress in PQQ production during fermentation, the fed-batch fermentation of mutant strain AE-9 was further investigated by combining different H_2_O_2_ feeding modes in a 3.7-L bioreactor. Modes A, B, and C were intermittent feeding of 100 mL H_2_O_2_ every 4 h after 32-h fermentation, with concentrations of 200 mM, 400 mM, and 600 mM, respectively (Fig. 4). As shown in Table 1, mode A brought a relatively high biomass (OD_600_ = 32.8) and PQQ accumulation (277 mg/L). Mode B presented the highest PQQ production (296 mg/L) and productivity (3.36 mg/L/h) compared with other feeding modes. Mode C gave the minimal biomass (OD_600_ = 29.0) and the lowest PQQ production (250 mg/L). As a result, the PQQ production was further increased by 16.3% with a suitable H_2_O_2_ inducement in the fermentation.

**Table 1:** PQQ production and productivity in fed-batch fermentation under different H_2_O_2_ treatments

Fed-batch modes	H_2_O_2_ (mM)	Period (h)	OD_600_	PQQ (mg/L)	PQQ productivity (mg/L/h)
A	200	88	32.8 ± 1.15^a^	277 ± 7.1^a^	3.2 ± 0.08^a^
B	400	88	34.7 ± 0.25^a^	296 ± 3.2^b^	3.4 ± 0.04^a^
C	600	88	29.0 ± 0.72^b^	250 ± 1.6^c^	2.8 ± 0.02^b^

Data shown as the mean ± SE of 3 replicates (*n* = 3); superscript alphabets (a, b, c) indicate statistically significant differences (*P* < 0.05)

**Fig. 5:**
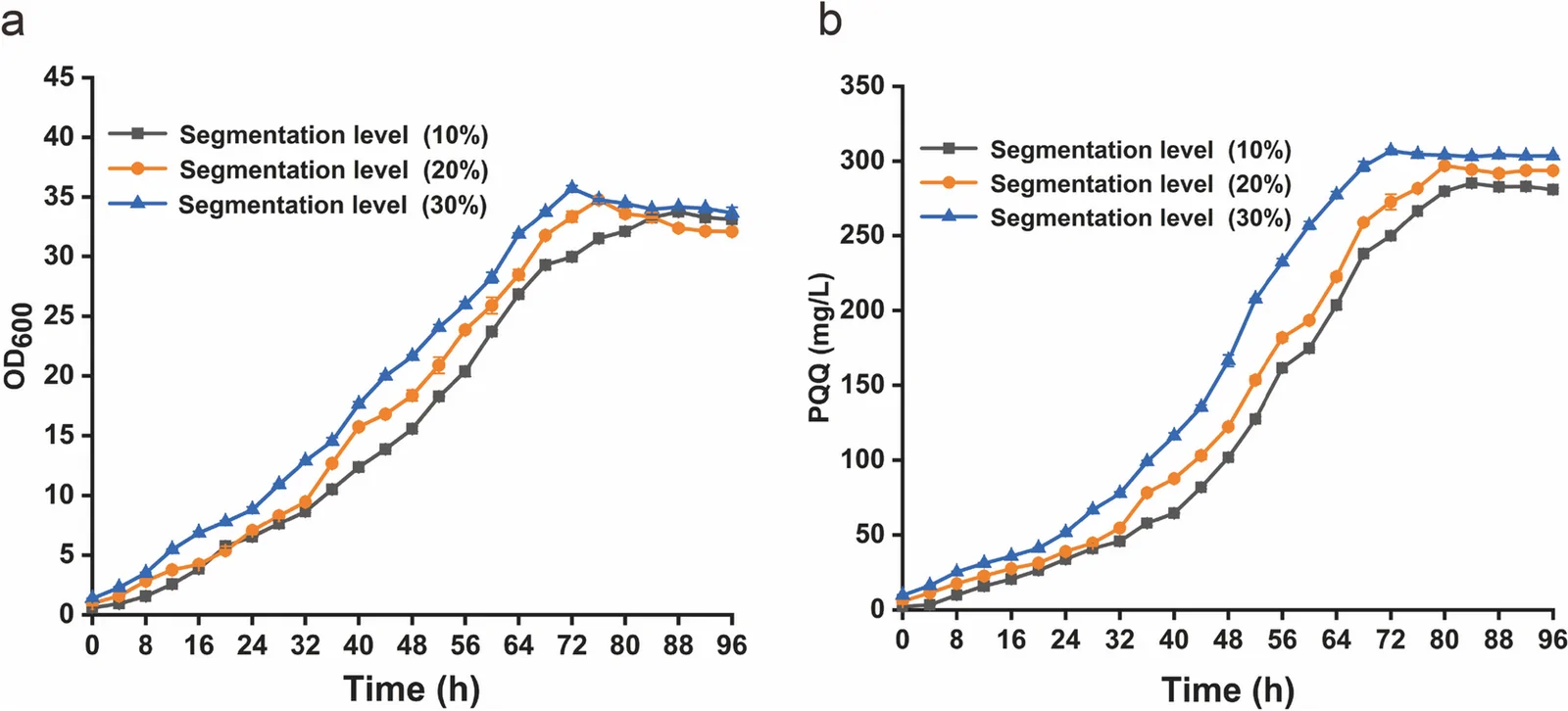
Cell growth (**a**) and PQQ production (**b**) in segmented fermentation of mutant strain AE-9 in a 3.7-L bioreactor

Furthermore, it has been reported that segmented fermentation has the advantages of rapidly initiating cell growth, separating the growth and product synthesis, and decreasing the fermentation cost (Wang et al. 2016). To decouple the growth of the AE-9 cells and the yield of PQQ, a segmented fermentation strategy was carried out by splitting the fermentation culture into the fresh medium with three different portions (10%, 20%, 30%). The results showed that biomass and PQQ yield increased with the increase of segmentation ratio (Fig. 5). When the split ratio was 30%, the AE-9 strain showed the highest biomass (OD_600_ = 35.7), highest PQQ production (307 mg/L), and PQQ productivity (4.26 mg/L/h) (Fig. 5).

### Genomic alterations in the mutant strain AE-9

Identification of mutations in the genome throughout the ALE and mutagenesis is important to understand the fitness of organisms under the desired condition. To address this, the mutant strain AE-9 and the wild strain H4-45 were proceeded for whole genome sequencing. Then, we aligned the mutant strain reads to the wild strain genome to search for mutations that might be related to the high production of PQQ. In total, seven single nucleotide variations (SNVs) in coding regions were identified in the mutant strain AE-9. As shown in Table 2, three SNVs were found in the *ftfL* gene, three were in the *thiC* gene, and one was in the 16 s rDNA. Of the seven SNVs, only one non-synonymous mutation occurred in the *ftfL* gene, leading to a change of Ala (GCC) to Pro (CCC) at position 485. The *ftfL* gene encodes a formate-tetrahydrofolate ligase (FtfL), which is involved in the methanol metabolism pathway by converting formate into formyltetrahydrofolate (Kang et al. 2020; Kim et al. 2020). Although it was not located at the active sites of the FtfL, the mutation was supposed to affect its ligation activity by replacing a small non-polar amino acid with a large polar amino acid. Besides, two synonymous SNVs occurred in the *ftfL* gene, and three synonymous SNVs occurred in the *thiC* gene (Table 2). The *thiC* gene encodes a thiamine biosynthesis protein. Although synonymous SNV does not alter the primary structure of the protein and can usually be considered a neutral event, some reports have shown that synonymous SNV may affect codon reading speed and alter the folding of the encoded protein (Jiang et al. 2023; Moreira-Ramos et al. 2022; Shen et al. 2022a). These results may explain the faster methanol consumption rate and the increase of growth in the mutant strain, which then leads to an enhanced PQQ production.

**Table 2:** List of identified single nucleotide variations in the mutant strain AE-9

Gene	Mutation	Amino acid change	Annotation
*ftfL*	1134C > G	Val(gtg)^378^Val(gtc)	Encoding the formate-tetrahydrofolate ligase
*ftfL*	1164G > A	Asp(gac)^388^Asp(gat)	Encoding the formate-tetrahydrofolate ligase
*ftfL*	1453C > G	Ala(gcc)^485^Pro(ccc)	Encoding the formate-tetrahydrofolate ligase
*thiC*	1419G > A	Pro(ccg)^473^Pro(cca)	Encoding thiamine biosynthesis protein
*thiC*	1437G > A	Ala(gcg)^479^Ala(gca)	Encoding thiamine biosynthesis protein
*thiC*	1440G > C	Pro(ccg)^480^Pro(ccc)	Encoding thiamine biosynthesis protein
16S rDNA	1340A > G	-	Encoding 16S rRNA

Additionally, an A to G transversion at position 1340 occurred in the 16S rDNA of the mutant strain. It has been reported that aminoglycoside antibiotics such as kanamycin bind to the A-site decoding region (helix 44) of the 16S rRNA to disturb the decoding process, and mutations of certain nucleotides in rRNA reduce aminoglycoside binding affinity (Recht et al. 1999; Ryu and Rando 2001). We speculate that the mutation in 16S rDNA might be linked to the tolerance of kanamycin in the mutant strain.

## Discussion

PQQ production has aroused strong commercial interest due to its antioxidant activity and capacity for scavenging free radicals. Some methanol-utilizing bacteria, especially *Hyphomicrobium denitrificans*, were screened out to accumulate PQQ with methanol as the carbon source (Urakami et al. 1992; Xiong et al. 2011). In fact, methanol has numerous advantages as a carbon source in fermentation processes, e.g., readily available, low market price, and easy to transport (Whitaker et al. 2015). However, the concentration and productivity of PQQ were relatively low in the wild strain. Therefore, systematic investigations on obtaining PQQ high-titer mutations and optimizing fermentation mode were necessary to improve PQQ production.

Random mutagenesis approaches coupled with adaptive laboratory evolution (ALE) strategies have been developed to enhance PQQ production in some methylotrophic bacteria. The *Methylobacterium extorquens* was employed for random mutagenesis by atmospheric and room temperature plasma (ARTP) to increase PQQ production from 8.6 to 16.02 mg/L (Li et al. 2018). Another *Methylobacterium* sp. strain was mutated by UV, NTG, EMS, and LiCl-UV for 11 consecutive generations, and the PQQ concentration increased from 11.21 to 19.33 mg/L (Zhao et al. 2021). By integrating ARTP mutagenesis and methanol tolerance of ALE, an *H. denitrificans* mutant strain was obtained to increase PQQ production from 29.6 to 69.5 mg/L (Ren et al. 2023). In this study, an *H. denitrificans* strain isolated by our lab was mutated by integrating UV-LiCl mutagenesis and ALE with oxidative stress, and the PQQ production increased from 17.14 to 30.96 mg/L. Despite the strain differences, PQQ production showed a similar growth rate between our study and other reports using different mutagenesis approaches and ALE methods.

In addition, many fermentation strategies have been employed to improve PQQ production. Optimization of the fed-batch fermentation process usually has a high efficiency in improving PQQ production. A two-stage pH control strategy was used to improve PQQ production to reach 353.28 mg/L in *Methylobacillus* sp. CCTCC M2016079 (Si et al. 2017). Besides, Liu et al. optimized a fermentation system with a two-stage oxygen supply strategy to increase PQQ production to 1070 mg/L in *H. denitrificans* FJNU-6 (Liu et al. 2020). In our study, the fermentation process was optimized with a pH control strategy, methanol feed flow, appropriate H_2_O_2_ treatment, and segmented fermentation, leading to PQQ concentration reaching 307 mg/L in the mutant strain AE-9. The PQQ production in this study is not the highest among different strains in the reports until now, but integration of random mutagenesis approaches and fermentation optimization strategies is efficient to obtain high-titer mutants and high concentration of target product, and the mutant strain AE-9 in this study has the potential of high PQQ production and deserved to be explored in the future.

The development of molecular biology and genetic manipulation has already been used to identify the PQQ biosynthetic gene cluster. Although the understanding of the PQQ biosynthetic pathway made it possible to achieve a high production of PQQ, only a limited increase was achieved by stimulating the expression of PQQ synthesis genes (Klinman and Bonnot 2014; Shen et al. 2012; Zhu and Klinman 2020). Mi et al. constructed a cassette with repetitive *tac* promoters to overexpress PQQ synthesis genes in *Klebsiella pneumoniae*, but PQQ production reached only 0.78 mg/L in a 5-L bioreactor (Mi et al. 2020). Until now, there is no report of PQQ high-yield *H. denitrificans* strain by genetic manipulation due to the insufficient genetic engineering tools and the uncleared metabolic network and regulation mechanism of PQQ biosynthesis. Our findings of genomic alterations in the *ftfL* gene may help to guide metabolic engineering in *H. denitrificans* to enhance methanol metabolism and PQQ production, which need to be verified in the future.

In conclusion, the present work showed that UV-LiCl mutagenesis and ALE technology were effective in obtaining a high-titer PQQ mutant strain *H. denitrificans* AE-9 by improving strain tolerance against oxidative stress. The mutant strain AE-9 showed an 80.4% increase in PQQ production and a 14.9% increase in cell density than the wild strain. Then fermentation optimization with combining pH-control strategy, feeding of methanol, H_2_O_2_ treatment, and segmented fermentation process was adopted to further enhance the production of PQQ. The highest PQQ production and productivity of the mutant strain AE-9 reached 307 mg/L and 4.26 mg/L/h in a 3.7-L bioreactor, respectively. Whole genome sequencing analysis showed mutations in *ftfL* and *thiC* genes that are involved in methanol and energy metabolism, which led to both increases in strain biomass and PQQ production. Our strategy used in this study might be applicable to improve the production of other compounds with antioxidant properties.

## Supplementary information

Below is the link to the electronic supplementary material.

Supplementary file1 (PDF 2817 KB)

## Data Availability

Please contact the corresponding author upon reasonable data requests.
