# Supplementary material for: Oxidative stress resistance prompts pyrroloquinoline quinone biosynthesis in Hyphomicrobium denitrificans H4-45
Source: Appl Microbiol Biotechnol. 2024 Feb 13;108:204. doi: 10.1007/s00253-024-13053-1 (PMC10864529; doi:10.1007/s00253-024-13053-1)
Supplement: Supplementary file 1 [file 253_2024_13053_MOESM1_ESM.pdf]

Supplementary data

Oxidative stress resistance prompts pyrroloquinoline quinone biosynthesis in *Hyphomicrobium denitrificans* H4-45

Jiale Liang[1], Mingjie Tang[1], Lang Chen[1], Wenjie Wang[1]*, Xinle Liang[1]*

[1] School of Food Science and Biotechnology, Zhejiang Gongshang University, Hangzhou, 210018, China

*Corresponding author.

Email: wenjiewang@mail.zjgsu.edu.cn (Wenjie Wang)

E-mail: dbiot@mail.zjgsu.edu.cn (Xinle Liang)

Phone: 86-571-8807-1024

Fax: 86-571-8820-6627

Table S1 All strains used in this study

Strains	Description	Sources
H. denitrificans H4-45	Wild type	This study
H. denitrificans AC-6	Best-performing isolate in the first stage of UV-LiCl mutagenesis and ALE	This study
H. denitrificans AD-17	Best-performing isolate in the second stage of UV-LiCl mutagenesis and ALE	This study
H. denitrificans AE-9	Best-performing isolate in the third stage of UV-LiCl mutagenesis and ALE	This study

Figure Legends

Fig. S1 Effects of different UV treatment times on the mortality of strain H4-45

Fig. S2 Determination of initial concentration of three screening stress in the three rounds of ALE：(**a**) Kanamycin, (**b**) Na_2S (**c**) K_2TeO_3

Fig. S3 Standard curve of PQQ measured by HPLC with different PQQ concentrations (9.4 mg/L, 18.8 mg/L, 37.5 mg/L, 75 mg/L, 150 mg/L, 300 mg/L)

Fig. S4 PQQ production of selected strains after the first stage of UV-LiCl mutagenesis and ALE

Fig. S5 PQQ production of selected strains after the second stage of UV-LiCl mutagenesis and ALE

Fig. S6 Genetic stability of mutant strain AE-9 after nine consecutive passages

Fig. S7 OD_{600} of mutant strain AE-9 in the MM medium with no pressure, full concentration of kanamycin+Na_2S+K_2TeO_3 treatment, and half concentration of kanamycin+Na_2S+K_2TeO_3 treatment

Fig. S8 Batch fermentation of PQQ production in a 3.7 L bioreactor without pH adjustment

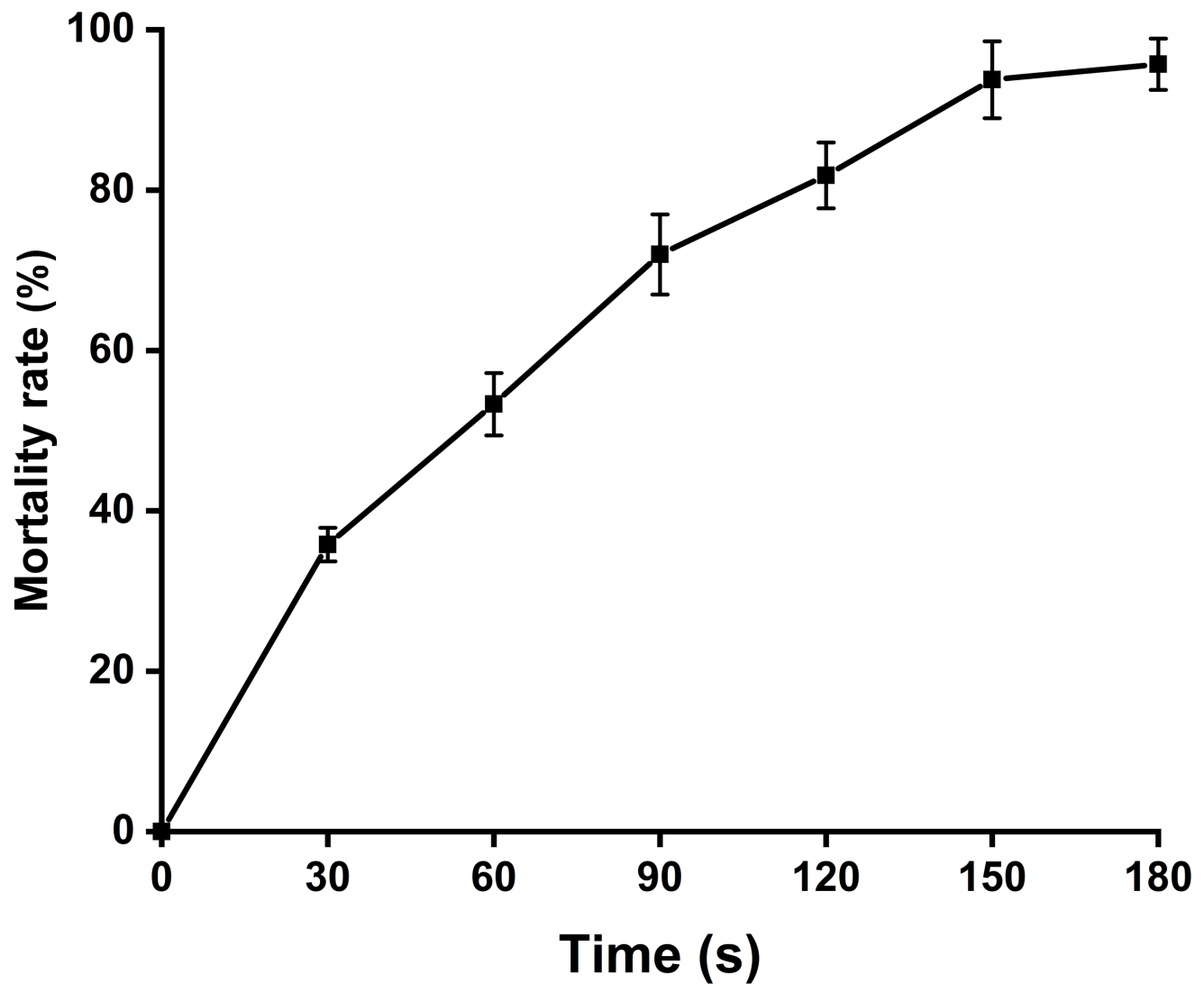

Fig. S1 Effects of different UV treatment times on the mortality of strain H4-45

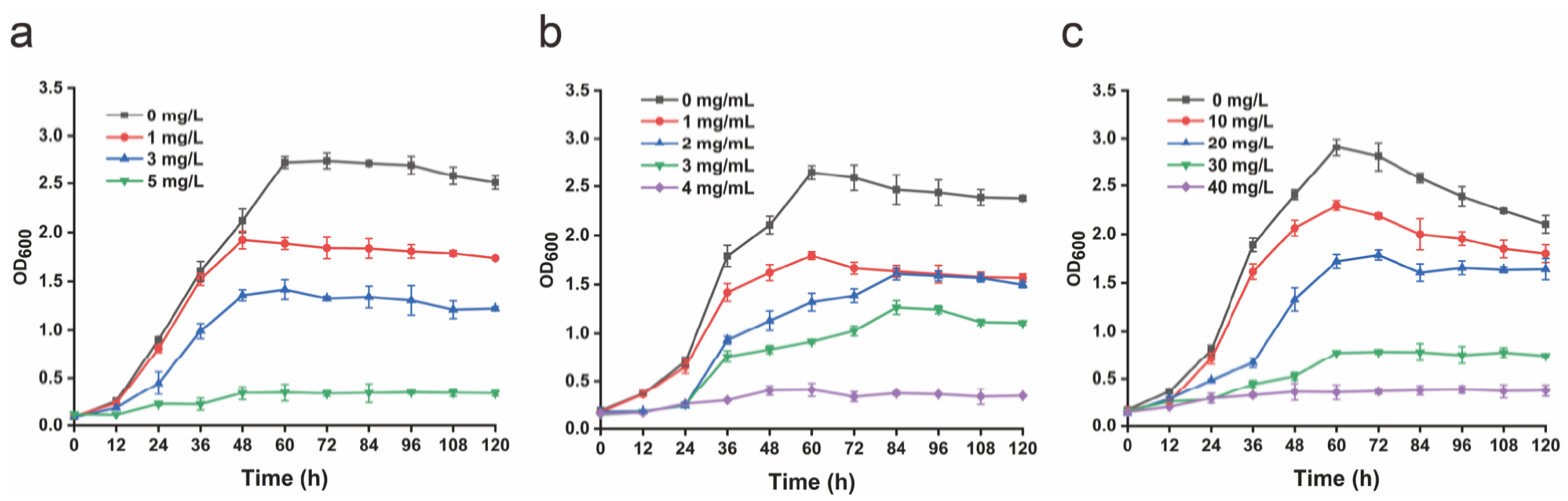

Fig. S2 Determination of initial concentration of three screening stress in the three rounds of ALE：(**a**) Kanamycin, (**b**) Na_2S (**c**) K_2TeO_3

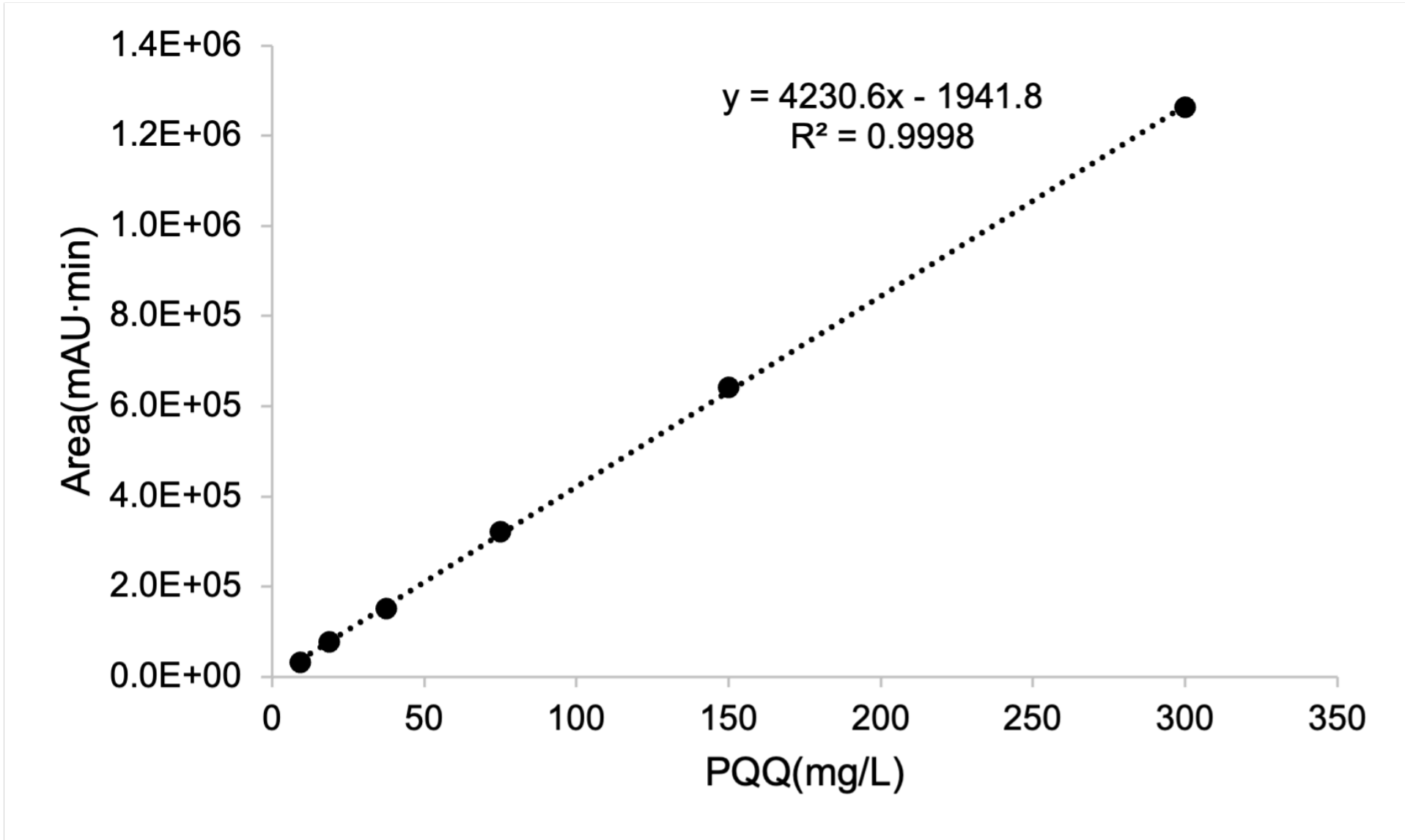

Fig. S3 Standard curve of PQQ measured by HPLC with different PQQ concentrations (9.4 mg/L, 18.8 mg/L, 37.5 mg/L, 75 mg/L, 150 mg/L, 300 mg/L).

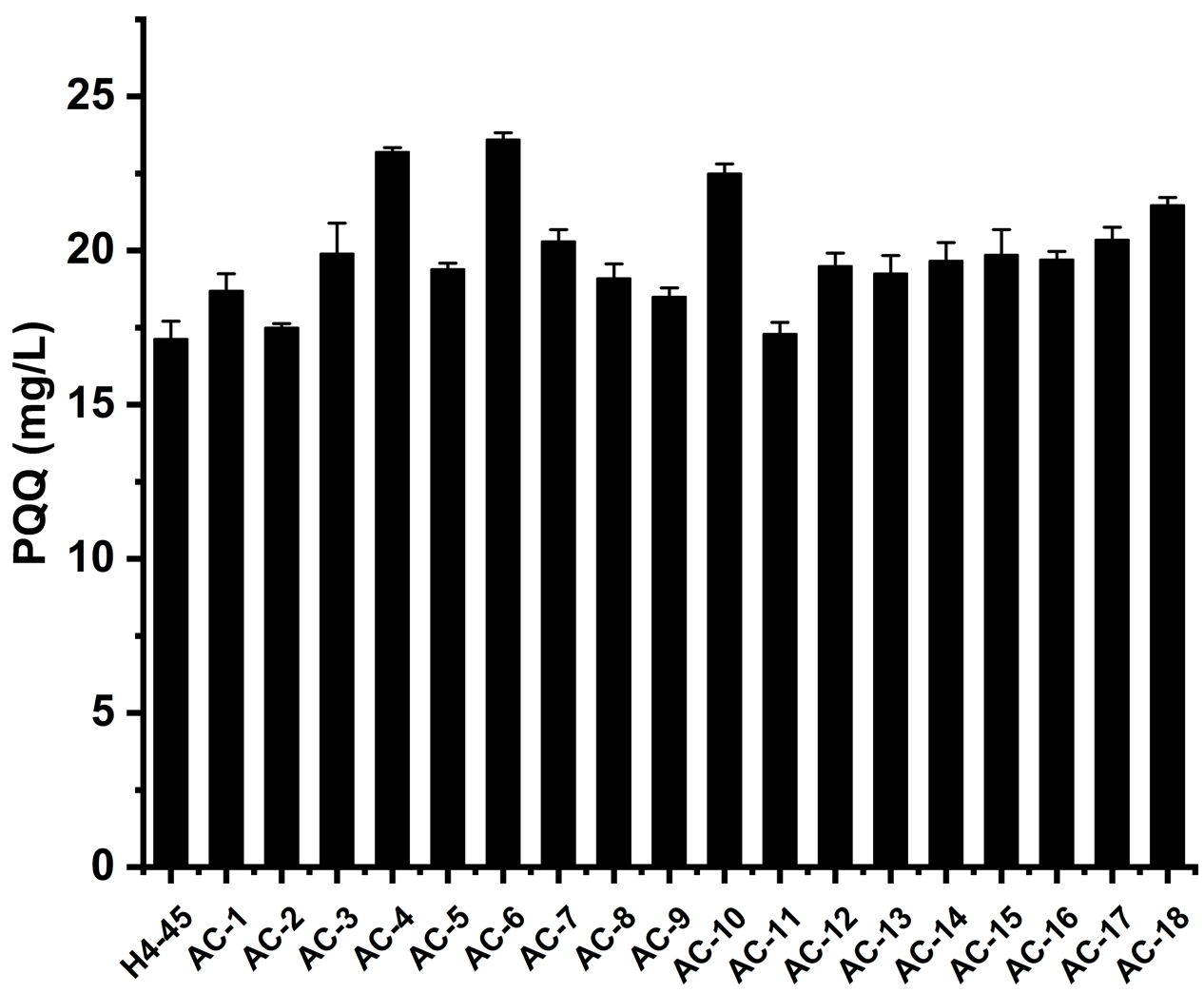

Fig. S4 PQQ production of selected strains after the first stage of UV-LiCl mutagenesis and ALE

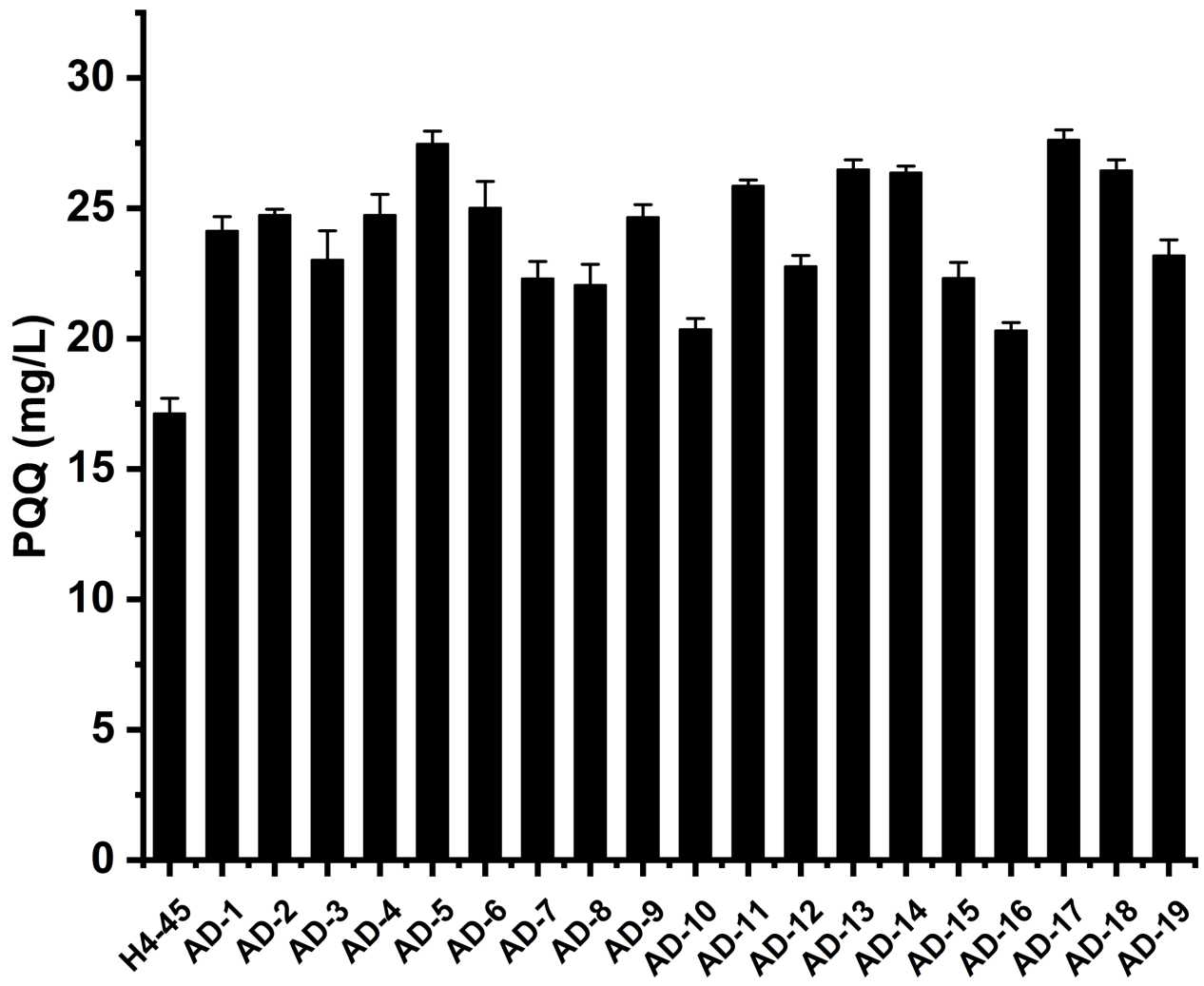

Fig. S5 PQQ production of selected strains after the second stage of UV-LiCl mutagenesis and ALE

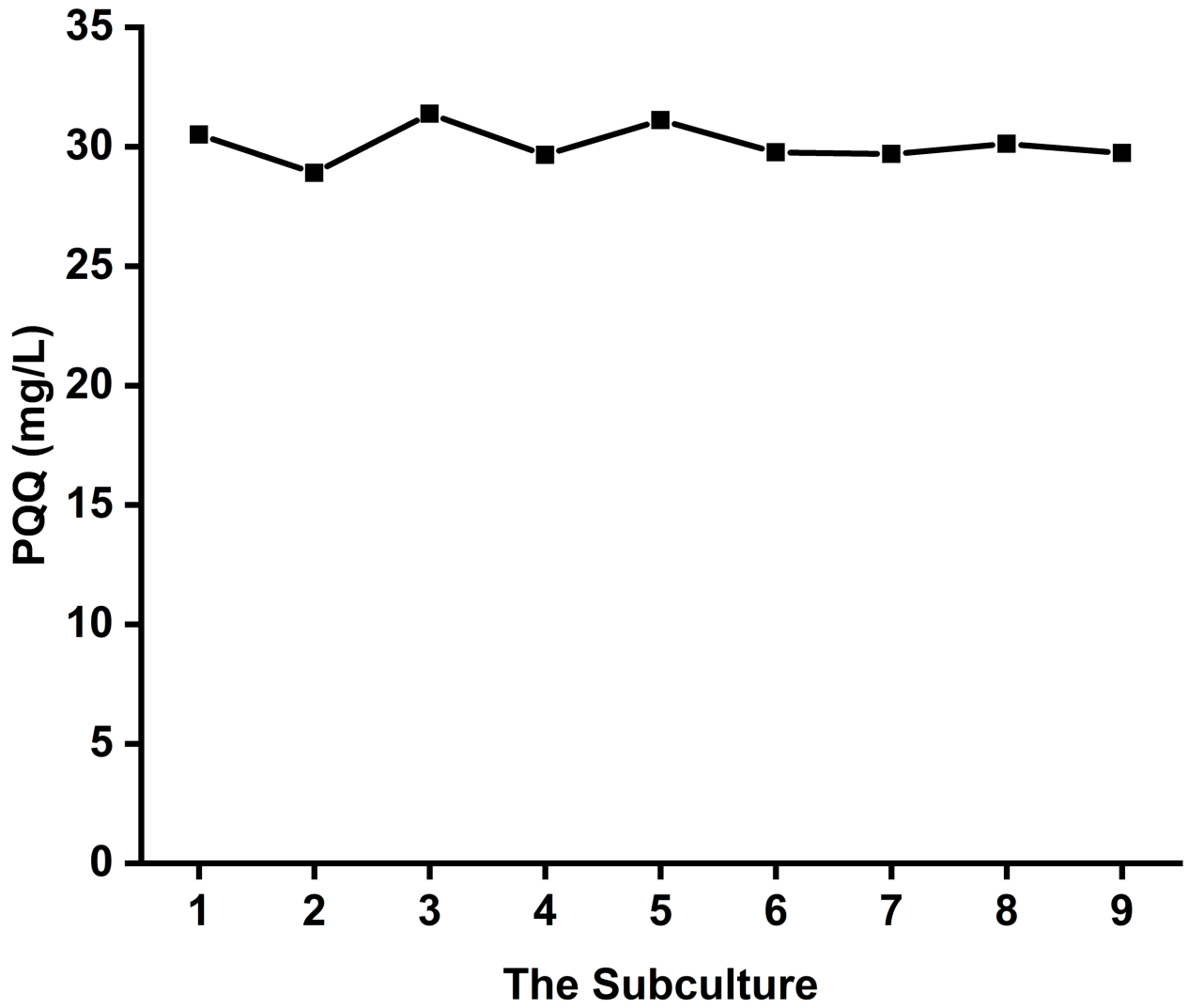

Fig. S6 Genetic stability of mutant strain AE-9 after nine consecutive passages

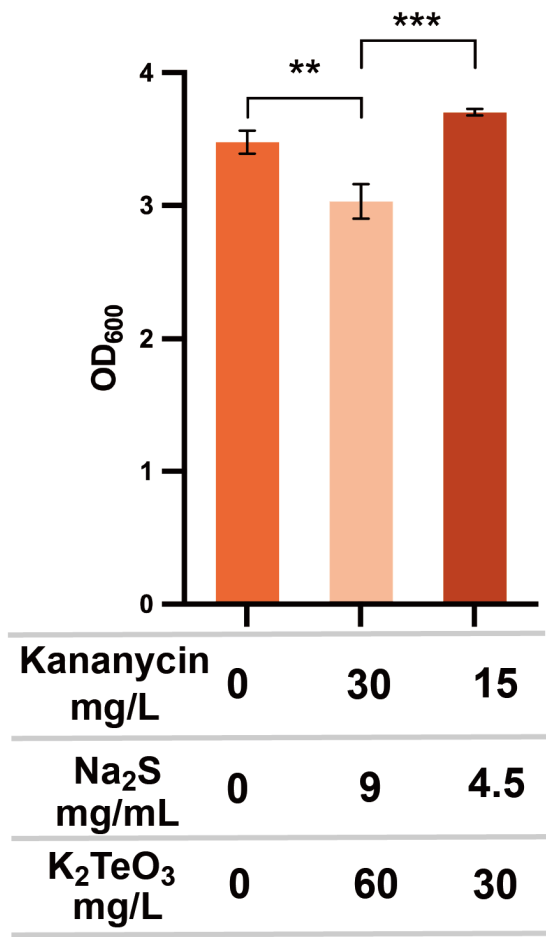

Fig. S7 OD_{600} of mutant strain AE-9 in the MM medium with no pressure, full concentration of kanamycin+Na_2S+K_2TeO_3 treatment, and half concentration of kanamycin+Na_2S+K_2TeO_3 treatment.

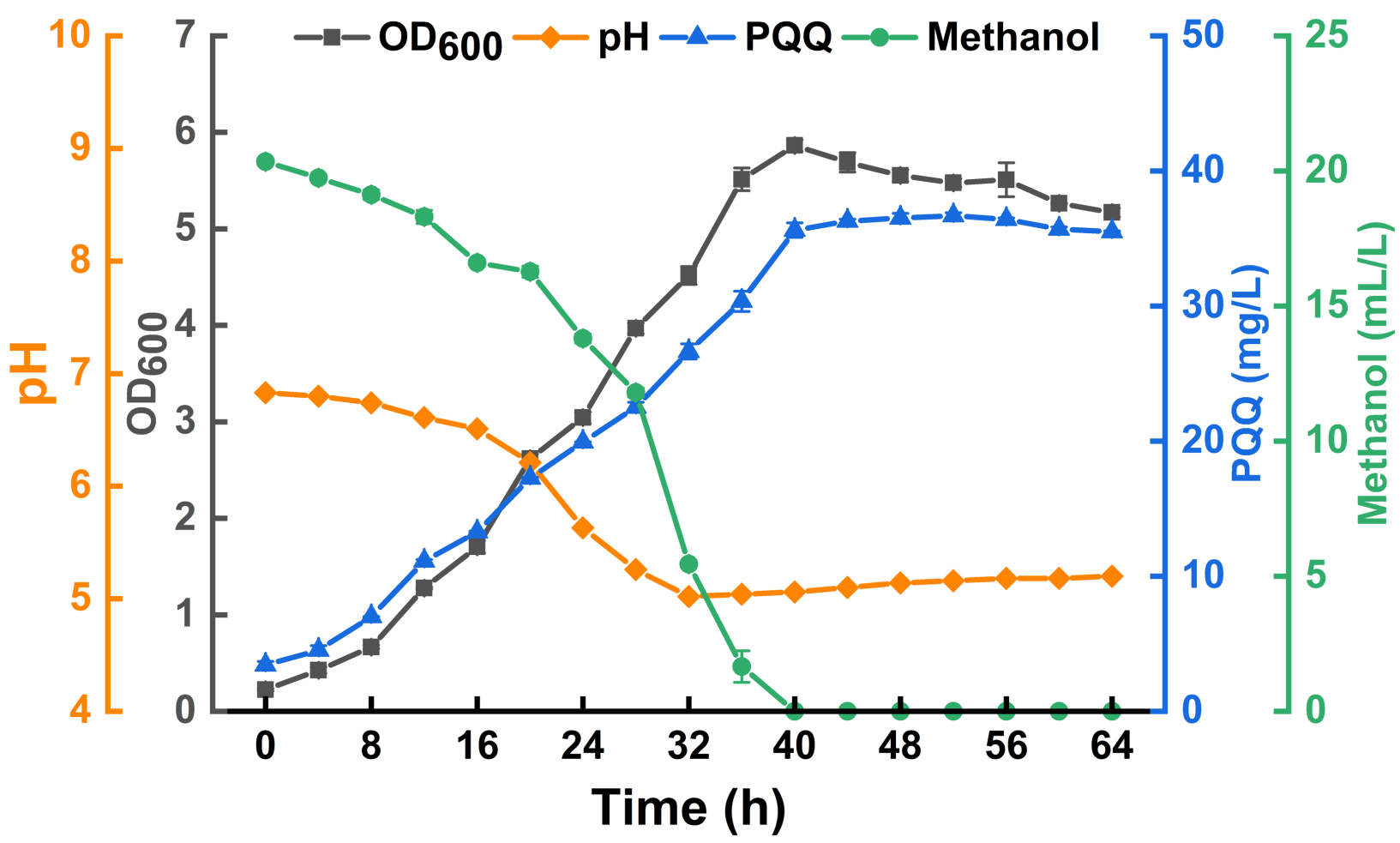

Fig. S8 Batch fermentation of PQQ production in a 3.7 L bioreactor without pH adjustment.
